# Transient anterior subcapsular vacuolar change of the crystalline lens in patients after posterior chamber phakic intraocular lens implantation

**DOI:** 10.1186/1471-2415-13-61

**Published:** 2013-10-25

**Authors:** Jin Kwon Chung, Jin Hee Shin, Sung Jin Lee

**Affiliations:** 1 Department of Ophthalmology, Soonchunhyang University Hospital, 59, Daesagwan-gil, Yongsan-gu, Seoul 140-743, Republic of Korea

**Keywords:** Implantable collamer lens, Phakic intraocular lens, Vacuolar change, Crystalline lens, Subcapsular opacities

## Abstract

**Background:**

We present two cases of transient vacuolar changes in the anterior subcapsular space of the crystalline lens in patients after posterior chamber phakic intraocular lens implantation.

**Case presentation:**

Implantable collamer lenses (ICL) were implanted in healthy myopic patients. Vacuolar changes developed just after the irrigating procedure through the narrow space between the ICL and the crystalline lens. Slit-lamp examinations and spectral domain optical coherence tomography showed bleb-like lesions in the anterior subcapsular space of one eye in each case, though the lesions gradually improved without visual deterioration. Consequently, the lesions turned into a few anterior subcapsular small faint opacities.

**Conclusion:**

Direct irrigation of the narrow space confined by the ICL and the crystalline lens is at risk for the development of vacuolar changes in the crystalline lens. The observed spontaneous reversal indicates that surgeons should not rush to surgical intervention but rather opt for close follow over several weeks.

## Background

Implantation of a posterior chamber phakic intraocular lens (IOL), such as, the implantable collamer lens (ICL; STAAR® Surgical AG, Monrovia, CA, USA), is a highly effective and safe procedure for the correction of high myopia with regard to immediate visual and refractive results [1,2]. As the phakic IOL is placed in the ciliary sulcus, the crystalline lens related complication could be developed. Cataract can be caused by the mechanical interaction and/or by an altered metabolism between the phakic IOL and the crystalline lens or by the surgical procedure [2,3]. The majority of ICL-associated cataracts have been reported as anterior subcapsular with an occurrence rate from 1.3 to 28% in the ICL for myopia (ICM) group [4].

We present two cases of vacuolar change in the anterior subcapsular space of a crystalline lens that developed during viscoelastics removal at the end of surgery and resolved spontaneously.

## Case presentation

### Case 1

**Figure 1:**
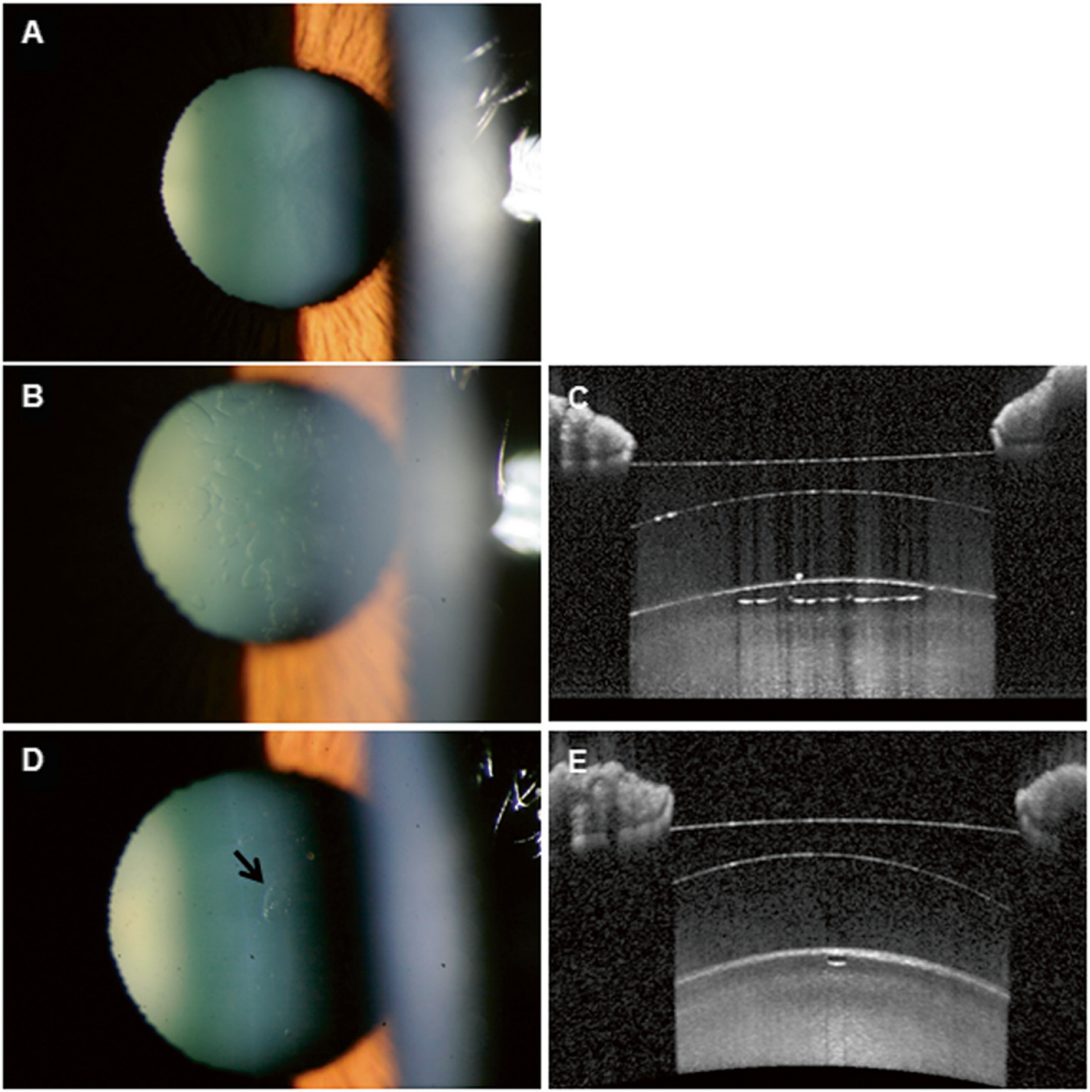
**Anterior segment photographs and optical coherence tomography findings in Case 1. A**: Slit-lamp examination showed a clear crystalline lens preoperatively. One day after ICL implantation, UDVA was 20/80 in the right eye. **B, C**: Slit-lamp examination showed bleb-like changes in the anterior subcapsular space and AS-OCT showed absence of reflected signal in the corresponding lesion. **D, E**: One year postoperatively, UDVA improved to 20/20 and a marked decrease of subcapsular vacuolar change (arrow) was observed with several small faint opacities.

A 38-year-old healthy man visited our clinic for ICL implantation. Preoperative corrected distance visual acuity (CDVA) was 20/20 in both eyes with refraction of -5.75–0.75 × 180° in the right eye and -5.75–1.25 × 170° in the left. Slit-lamp and fundus examinations were unremarkable (Figure 1A). Anterior chamber depths (ACD) in right and left eyes were 2.91 and 2.96 mm, respectively.

The calculated ICL models were 12.5 mm, ICMV4 -9.00 diopters (D) and 12.5 mm, toric ICMV4 -9.50 +1.00 90°. Briefly, topical anesthesia was administered, paracentesis was made, and viscoelastic material (Discovisc®, Alcon Laboratories, TX, USA) was used for the surgery. The ICL was injected through a temporal clear corneal incision and each footplate in turn was placed beneath the iris without placing pressure on the crystalline lens. As the final step, viscoelastic material was removed by gentle irrigation using balanced salt solution (BSS). The surgeon (J.K.C) tried to remove small air bubble between the ICL and the crystalline lens, by placing a blunt cannula close to the gap between ICL optic and the crystalline lens, and then performing gentle irrigation. However, an anterior subcapsular vacuolar change was identified at the end of the procedure. Uneventful toric ICL implantation was then performed in the left eye.

The day after surgery, uncorrected distance visual acuities (UDVA) in the right and left eyes were 20/80 and 20/20, respectively. Slit-lamp examination showed bleb-like lesions of the anterior lens capsule of the right eye (Figure 1B) and spectral domain anterior segment optical coherence tomography (AS-OCT, Heidelberg Engineering, Carlsbad, CA, USA) demonstrated anterior subcapsular vacuolar change of the crystalline lens (Figure 1C). No cataractous change was observed in the anterior or posterior lens cortex or nucleus of the right eye. Vaulting was measured at 512 um by AS-OCT and the intraocular pressure (IOP) was 19 mmHg. Because postoperative examinations showed different changes in the appearance of the traumatic cataract and normal vaulting, the patient was managed with routine postoperative medication, which included moxifloxacin and prednisolone eye drops 4 times daily. The sizes and numbers of vacuolar changes started to disappear spontaneously at 2 weeks after surgery and UDVA improved to 20/32. At one year postoperatively, UDVA was 20/20 in the right eye and the lesion had noticeably decreased. Although there was left faint opacity in the right eye, no further progression of the cataract has been observed (Figures 1D, E).

### Case 2

A 30-year-old healthy woman visited our clinic for ICL implantation. CDVA was 20/20 in both eyes with refractions of -3.00–0.50 ×180° and -2.75–0.50 ×165° in right and left eyes, respectively. Slit-lamp and fundus examinations were unremarkable. ACDs were measured at 2.99 and 3.02 mm in right and left eyes, respectively.

The calculated ICL models were 11.5 mm, ICMV4 -5.00 D and 12.5 mm, ICMV4 -4.5 D. The patient received the same procedure as described for case 1 by another surgeon (S.J.L). The only difference being superior clear corneal incision. Uneventful ICL implantation was performed first in the right eye. However, as occurred in Case 1, during the final step of surgery on the left eye, while viscoelastics between the lens were removed by gentle irrigation with BSS, vacuolar changes were generated in the anterior subcapsular space of the crystalline lens.

**Figure 2:**
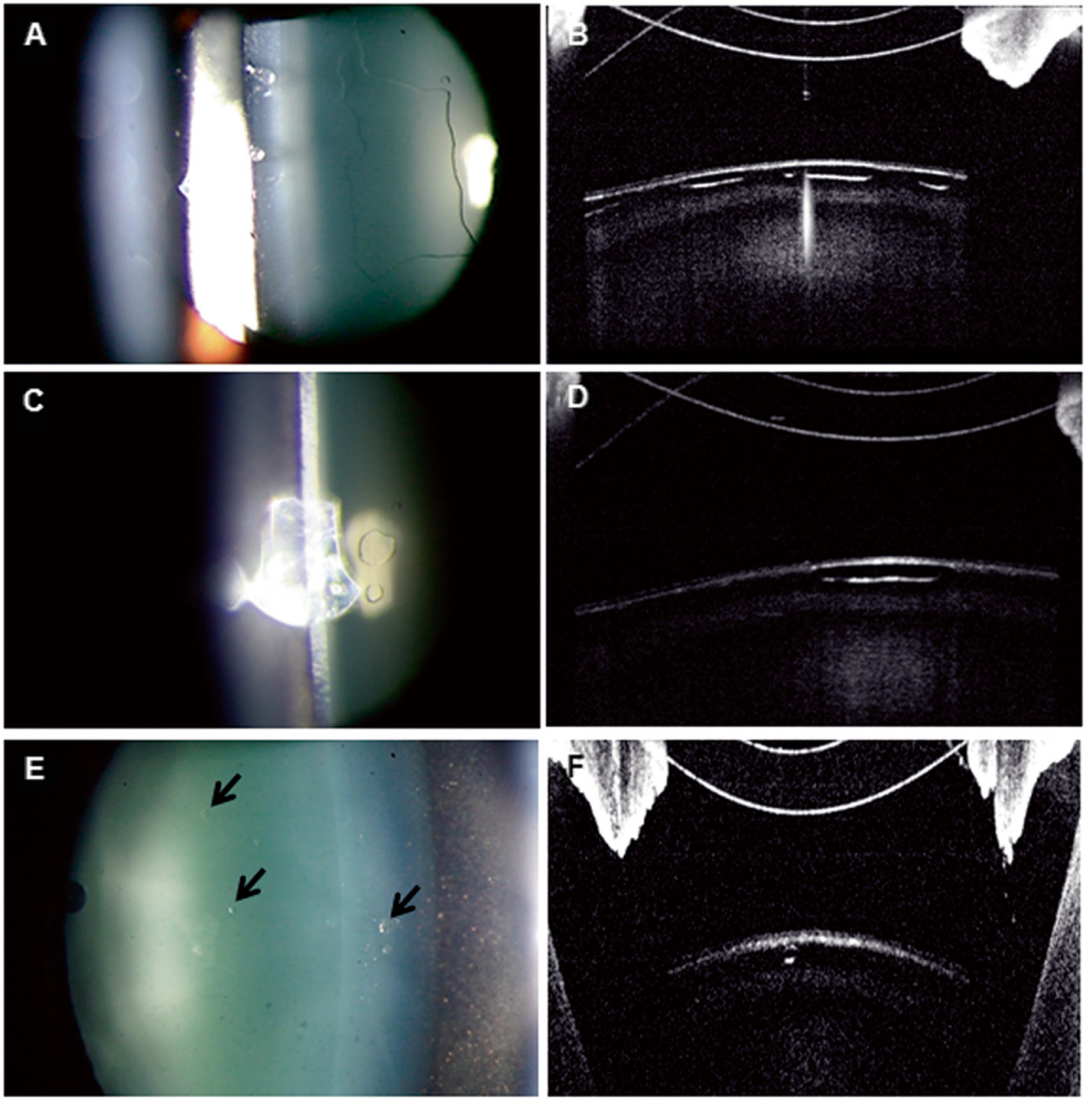
**Anterior segment photographs and optical coherence tomography findings in Case 2.** One day after ICL implantation, UDVA was 20/20 in the left eye. **A, B**: Slit-lamp examination showed a large bleb-like change in the anterior subcapsular space and AS-OCT showed the absence of a reflected signal in the corresponding lesion. **C, D**: Three months after ICL implantation, a marked decrease in the subcapsular vacuolar change was observed. **E, F**: At 6 months after surgery, the vacuolar lesion showed small faint opacities, which were not clinically significant, and UDVA remained at 20/20.

The day after surgery, UDVA was 20/20 in the left eye, and a slit-lamp examination showed bleb-like lesions in the anterior subcapsular space of the crystalline lens (Figure 2A) and AS-OCT showed vacuolar change just beneath the anterior capsule, as was observed in Case 1 (Figure 2B). Vaulting was measured at 755 um by AS-OCT and IOP was 17 mmHg in the left eye. Nevertheless, lesions started to disappear spontaneously at 1 week after surgery and UDVA was maintained at 20/20. After 3 months, subcapsular vacuoles decreased markedly (Figures 2C, D), and at 6 months, only several faint subcapsular opacities remained (Figures 2E, F). No further progression of cataract in the cortex or nucleus of the lens has been observed.

## Discussion and conclusion

Crystalline lens opacification after ICL implantation may have a multifactorial etiology, and intraoperative trauma, contact between the ICL and the crystalline lens, and perioperative inflammation have been reported to cause crystalline lens opacification [5]. Anterior subcapsular cataract is the most common cataract type of the ICL-associated cataracts [2-4], and induces epithelial deterioration [6,7]. Furthermore, lens epithelial cells do not have reparative ability [8], and thus, the opacification is permanent.

In our cases, subcapsular bleb-like lesion gradually improved, however there left small faint opacity. As postoperative vaulting was sufficient and perioperative inflammation was not significant, furthermore the vacuolar changes developed immediately additional irrigation procedure between the lenses so that the vacuolar change seems to be responsible for the residual crystalline lens opacities. This type of traumatic cataract should be added to the possible complications, including previously reported traumatic cataract in eyes with ICL implantation.

One possible explanation is that the friction drag on the crystalline lens capsular surface during irrigation procedure was responsible for the lesion formation [9]. We tried to irrigate the narrow space with BSS in both cases, and we believe that the BSS flow, in accord with Bernoulli’s theorem, generated stress between the posterior surface of ICL and anterior capsule of the crystalline lens. Furthermore, BSS flow was retarded by the fixed anterior lens surface, and presumably, this resulted in a boundary layer and turbulence, which was created sufficient friction drag on the anterior lens surface to create the lesions. It is well known that during modern cataract surgery, the lens capsule and lens cortex can be easily separated during capsulorrhexis and hydrodissection because the capsule originates as the basement membrane of the epithelial cells of the embryonic lens vesicle [10]. We speculate that the subcapsular vacuolar changes resulted from multiple localized separations of the anterior lens capsule and the cortex caused by friction drag during BSS irrigation. The absence of a reflected signal in the lesions by AS-OCT supports this explanation.

We used Discovisc® as an ophthalmic viscosurgical device (OVD) instead of a cohesive low-viscous OVD, such as hydroxypropyl methylcellulose which is recommended by the STAAR surgical. This could be considered as another cause of the lesions because of the different physical and chemical properties. However, the lesions did not develop during all procedures of ICL implantation but developed immediately after the irrigation procedure so that it seems not to be related the lesions.

Although vacuolar changes gradually improved without visual deterioration, lesions had not completely disappeared at 1-year postoperatively. We surmise anterior lens capsular separation was accompanied by epithelial cell damage and that remnant vacuoles caused small faint opacities.

We believe that this is the first report of transient vacuolar changes in the anterior subcapsular space of the crystalline lens in patients after ICL implantation, and recommend that this be considered a possible complication. To avoid this complication meticulous care is required during irrigation procedure and low-viscous OVD free from trabecular meshwork occlusion should be used according to the manufacturer’s recommendation. Furthermore, because of the reversibility of this phenomenon, immediate intervention, such as, ICL removal and/or cataract surgery also should be sublated.

## Consent statement

Written informed consent was obtained from the patient for publication of this case report and any accompanying images. A copy of the written consent is available for review by the Editor of this journal.

## Competing interests

The authors declare that they have no competing interests.

## Authors’ contributions

JKC: patient interaction, diagnosis, performed surgery, and drafting manuscript. JHS: participated in information gathering, literature search, and drafting manuscript. SJL: patient interaction, diagnosis, performed, surgery, and data analysis. All authors read and approve the final manuscript.

## Pre-publication history

The pre-publication history for this paper can be accessed here:

http://www.biomedcentral.com/1471-2415/13/61/prepub
